# Natural killer (NK) cells inhibit systemic metastasis of glioblastoma cells and have therapeutic effects against glioblastomas in the brain

**DOI:** 10.1186/s12885-015-2034-y

**Published:** 2015-12-24

**Authors:** Se Jeong Lee, Won Young Kang, Yeup Yoon, Ju Youn Jin, Hye Jin Song, Jung Hyun Her, Sang Mi Kang, Yu Kyeong Hwang, Kyeong Jin Kang, Kyeung Min Joo, Do-Hyun Nam

**Affiliations:** 1 Department of Anatomy and Cell Biology, Sungkyunkwan University School of Medicine, 2066, Seobu-ro, Jangan-gu, Suwon-si, Gyeonggi-do 16419 South Korea; 2 Department of Neurosurgery, Samsung Medical Center, Sungkyunkwan University School of Medicine, 50 Ilwon-Dong, Gangnam-Gu Seoul, 06351 South Korea; 3 Department of Health Sciences and Technology, SAIHST, Sungkyunkwan University, 50 Ilwon-Dong, Gangnam-Gu Seoul, 06351 South Korea; 4 Cell Therapy Team, Mogam Biotechnology Institute, Yongin, 16928 South Korea; 5 Department of Anatomy and Cell Biology, Sungkyunkwan University School of Medicine, 50 Ilwon-Dong, Gangnam-Gu Seoul, 06351 South Korea

**Keywords:** Glioblastoma multiforme, Natural killer cell, Systemic metastasis, Orthotopic xenograft model, Therapeutic effect

## Abstract

**Background:**

Glioblastoma multiforme (GBM) is characterized by extensive local invasion, which is in contrast with extremely rare systemic metastasis of GBM. Molecular mechanisms inhibiting systemic metastasis of GBM would be a novel therapeutic candidate for GBM in the brain.

**Methods:**

Patient-derived GBM cells were primarily cultured from surgical samples of GBM patients and were inoculated into the brains of immune deficient BALB/c-nude or NOD-SCID IL2Rgamma^null^ (NSG) mice. Human NK cells were isolated from peripheral blood mononucleated cells and expanded in vitro.

**Results:**

Patient-derived GBM cells in the brains of NSG mice unexpectedly induced spontaneous lung metastasis although no metastasis was detected in BALB/c-nude mice. Based on the difference of the innate immunity between two mouse strains, NK cell activities of orthotopic GBM xenograft models based on BALB/c-nude mice were inhibited. NK cell inactivation induced spontaneous lung metastasis of GBM cells, which indicated that NK cells inhibit the systemic metastasis. In vitro cytotoxic activities of human NK cells against GBM cells indicated that cytotoxic activity of NK cells against GBM cells prevents systemic metastasis of GBM and that NK cells could be effective cell therapeutics against GBM. Accordingly, NK cells transplanted into orthotopic GBM xenograft models intravenously or intratumorally induced apoptosis of GBM cells in the brain and showed significant therapeutic effects.

**Conclusions:**

Our results suggest that innate NK immunity is responsible for rare systemic metastasis of GBM and that sufficient supplementation of NK cells could be a promising immunotherapeutic strategy for GBM in the brain.

**Electronic supplementary material:**

The online version of this article (doi:10.1186/s12885-015-2034-y) contains supplementary material, which is available to authorized users.

## Background

Glioblastoma multiforme (GBM) is the most common primary malignancy of the central nervous system (CNS), and 75 % of affected patients die within two years of their diagnosis [1–3]. GBMs are characterized by their highly infiltrative nature, which causes difficulties in curative surgical resection. Moreover, the resistance of GBM cells to radio- and chemo-therapy provokes a high rate of tumor recurrence [2, 4, 5]. Therefore, unmet medical need new therapeutic modalities with novel treatment mechanisms targeting GBM cells that evade and/or withstand currently available therapies.

Although GBMs are known as highly invasive tumors in the brain, extra-cranial metastasis does rarely occur [6–9]. This clinical characteristic of GBMs could be due to several reasons; short survival length of GBM patients, unique structure of micro-vessels in the CNS, lack of lymphatic systems in the CNS, and immune-privileged microenvironments [10–12]. The underlying mechanisms of the limited systemic metastatic potential of GBM cells are not only interesting from a scientific perspective, but could also provide clues leading to novel therapeutic modalities and unique treatment mechanisms.

Recently, orthotopic GBM xenograft animal models using patient-derived GBM cells have been utilized to test newly developed therapeutic agents of GBMs [13]. The animal models maintain the genetic, molecular, and functional features of the parental tumors to provide reliable preclinical models for GBMs. Since the xenograft models utilize immune deficient mouse strains to avoid graft rejection, immunologic microenvironments of transplanted GBM cells can be specifically modified by choosing a recipient mouse strain with defined immune deficiency status. For example, the BALB/c-nude strain has an innate immune system but no acquired immunity, while both the innate and acquired immune systems of the NOD-scid IL2Rgamma^null^ (NSG) mouse are impaired [14–16].

In this study, we elucidated a possible mechanism regarding the limited systemic metastatic potential of GBM cells in the brain using an orthotopic xenograft animal model in addition to discovering some anti-cancer activities of systemic NK cells. Since the brain microenvironments prevent GBM cells from having direct contact with NK cells, direct or indirect NK cell supplementation to the GBMs demonstrated significant therapeutic effects, in our preclinical model.

## Methods

### Cell culture

All Human samples were collected with written informed consent under a protocol approved by the Institutional Review Board of the Samsung Medical Center (2010–04–004, Seoul, Korea). Parts of the surgical samples were enzymatically dissociated, and then red blood cells were removed by percoll gradient centrifugation (Sigma-Aldrich). Dissociated cells were maintained in the ‘NBE’ conditions consisting of Neuro-Basal Media, N2 and B27 supplements (×1/2 each), 2 mM L-glutamine, 100U/ml penicillin and streptomycin (Invitrogen), and human recombinant EGF and bFGF (50 ng/ml each; R&D Systems). The human GBM cell line U-87 MG (ATCC) was maintained in Dulbecco's Modified Eagle Medium (DMEM) supplemented with 10 % fetal bovine serum, 100U/ml penicillin and streptomycin (Invitrogen).

### Patient-derived GBM xenograft model

All animal experiments were approved by the Institutional Review Boards of the Samsung Medical Center (Seoul, Korea) and conducted in accord with the ‘Health Guide for the Care and Use of Laboratory Animals’ (NIH publication no. 80–23) and the ARRIVE guidelines for Reporting Animal Research [17] (Additionanl file 1). For orthotopic GBM xenograft models, anesthetized 6-week-old BALB/c-nude or NOD-SCID IL2Rgamma^null^ (NSG) mice were secured in a rodent stereotactic frame (mice were obtained from Orient Bio Korea). A hollow guide screw was implanted into a small drill hole made at 2 mm left and 1 mm anterior to the bregma, and then 2 × 10^5^ tumor cells in 5 μl HBSS were injected through this guide screw into the white matter at a depth of 2 mm [anterior/posterior (AP) +0.5 mm, medial/lateral (ML) +1.7 mm, dorsal/ventral (DV) -3.2 mm]. Mice with a total body weight reduction >20 % were sacrificed, and their brains and lungs were processed for paraffin sections.

### Treatment with anti-asialo GM1 (ASGM1) antibody

Male 6-week-old BALB/c-nude mice were injected intravenously with the ASGM1 antibody (Wako Chemicals) or 1× Phosphate Buffered Saline (PBS, Invitrogen) on Day -1 (40 μl/ea). On Day 0, patient-derived GBM cells (2 × 10^5^) were implanted into the brain as described previously. After the tumor cell inoculation, either ASGM1 antibody or 1× PBS were intravenously injected into the animals twice a week for 4.5 months. Eighteen weeks after the tumor cell inoculation, the mice were sacrificed. Spleens were harvested and measured NK cells activity. Brains and lungs were paraffin-embedded, and then sliced into 4 μm sections for histological analysis.

For murine NK cell activity measurement, the spleens were immersed in Hank's Balanced Salt Solution (HBSS, Invitrogen), and then single cell suspensions were prepared by forcing the spleens through a 70 μm nylon mesh. The resulting cell suspension was placed onto a Ficoll-Paque PLUS (GE Healthcare) and centrifuged for 30 min at 2,000 rpm. Mononuclear cells were isolated, washed, and stained with a PE-Cy7-conjugated anti-mouse CD314 (NKG2D) antibody (eBioscience).

### Immunohistochemistry

Paraffin-embedded tissue sections were deparaffinized and rehydrated. Heat-induced epitope retrieval was performed using a target retrieval solution (Dako) for 5 min in a microwave. Slides were treated with 3 % hydrogen peroxide for 10 min to inactivate endogenous peroxidase, and then the slides were blocked for 20 min at room temperature in a blocking solution (5 % normal horse serum, 1 % normal goat serum, 0.1 % Triton-X 100 in 1× PBS). After blocking, the slides were incubated in primary antibodies at 4 °C overnight; including mouse monoclonal antibody against human Ki-67 (BD Pharmingen), mouse monoclonal antibody against human cytoplasm (STEM-121, Stem Cells), mouse monoclonal antibody against human nestin (Thermo), mouse monoclonal antibody against human SOX2 (Cell Signaling technology), mouse monoclonal antibody against human GFAP (Sigma), mouse monoclonal antibody against NK1.1 (Novus biological), and rabbit monoclonal antibody against human HLA-A (MHC class I, abcam). Slides were washed and incubated with secondary antibodies for 1 h at room temperature [Avidin-Biotin complex kit (Vector lab) or Alexa Flour 488 or 594 conjugated antibodies (Invitrogen)]. Slides were counterstained with hematoxylin (Sigma-Aldrich) or DAPI (Sigma-Aldrich).

### Western blotting and flow cytometry

Cell lysates were prepared using lysis buffer (50 mM HEPES, pH7.5, 150 mM NaCl, 1.5 mM MgCl_2_, 1 % Triton X-100, 10 % glycerol, and protease/phosphatase inhibitors; Roche). Protein concentrations were determined using a BCA protein assay kit according to the manufacturer's directions (Thermo). Equivalent amounts of proteins were separated by 10 % SDS gel electrophoresis, transferred onto PVDF membranes (Thermo), and immunoblotted with primary antibodies overnight at 4 °C, including a rabbit monoclonal antibody against human HLA-A (MHC class I, abcam) and a rabbit monoclonal antibody against GAPDH (Cell Signaling technology). Antibodies were visualized using a horseradish peroxidase-conjugated anti-rabbit IgG (Thermo) and analyzed using enhanced chemiluminescence western blot detection reagent (GE Healthcare).

For flow cytometry, anti-HLA-ABC-PE (G46-2.6), anti-MIC-A/B-PE (6D4) (BD Biosciences), anti-ULBP-1-PE (170818), anti-ULBP-2-PE (165903) (R&D systems), anti-HLA-E-PE (3D12), anti-CD112-PE (TX31), and anti-CD155-PE (SKII.4) antibody (BioLegend) were utilized. Samples were run on a BD Fortessa (BD Biosciences) and data were analyzed using FlowJo software (TreeStar Inc., OR).

### Human NK cell preparation and in vitro expansion

In vitro expansion of human NK cells was conducted as previously described [18]. Briefly, peripheral blood mononucleated cells (PBMCs) were isolated from healthy donors by leukapheresis and CD3^+^ T cells were depleted by VarioMACS (Miltenyi Biotech). T cell-depleted PBMCs were expanded at a seeding concentration of 2 × 10^5^ cells/ml in CellGro SCGM serum-free medium (CellGenix) with 1 % autoplasma, 1 × 10^6^ cells/ml irradiated (2,000 rad) autologous PBMCs, 10 ng/ml anti-CD3 monoclonal antibody, OKT3 (Orthoclon), and 500 IU/ml IL-2 (Proleukin) in an A-350 N culture bag (NIPRO). On day 5 of culture, NK cells were fed with fresh medium containing 500 IU/ml IL-2 and 1 % autoplasma every two days without removal of preexisting culture medium to maintain a cellular concentration at 1 ~ 2 × 10^6^ cells/ml for 14 days. The viability of expanded NK cells was determined by propidium iodide staining.

In vitro expanded NK cells were stained with primary antibodies and analyzed by a flow cytometry using anti-CD56-PE-Cy5 (B159), anti-CD16-PE (3G8), anti-CD3-FITC (UCHT1), anti-NKp30-PE (P30-15), anti-NKp44-PE (P44-8.1), anti-NKp46-PE (9E2/NKp46), anti-DNAM-1-PE (DX11), anti-CD14-FITC (M5E2), anti-CD19-PE (HIB19) (BD Biosciences), anti-NKG2D-PE (149810) (R&D systems), anti-CD158a-PE (EB6Bf), anti-CD158b-PE (GL183), and anti-CD158e-PE (Z27.3.7) (Beckman Coulter).

### ^51^Cr-release cytotoxicity assay

Target cells were labeled with 100 μCi ^51^ Cr sodium chromate (BMS), and incubated with NK cells in U-bottom 96-well plates (BD falcon) at three different effector:target (E:T) ratios. Spontaneous and maximum releases were determined by incubating target cells without effector cells in the absence or presence of 4 % Triton X-100. Radioactivity was counted using a gamma counter (PerkinElmer), and the percentage of specific lysis was calculated as follows: % specific lysis = [(experimental release–spontaneous release)/(maximum release-spontaneous release)] × 100. The assay was performed in triplicate.

### In vivo anti-tumor activities of NK cells

Orthotopic GBM xenograft models were established as described previously, using 2 × 10^5^ U-87 MG cells in 5 μl HBSS were injected in male 6-week-old BALB/c-nude mice brains. Human NK cells were injected intratumorally (1 × 10^3^, 1 × 10^4^, 1 × 10^5^ in 5 μl HBSS) or intravenously (1 × 10^5^, 1 × 10^6^, 1 × 10^7^, in 100 μl HBSS) into animals once a week for 3 weeks or 3 times a week for 1 week. After 28 days, the animals’ brains were harvested and cut into 4–6 mm thick slices.

Brain slices were fixed in 4 % paraformaldehyde, embedded in paraffin, sectioned into 4 μm coronal sections using a microtome, and stained with hematoxylin and eosin (Sigma). The tumor volume was calculated by measuring the section with the largest tumor portion and applying the formula: (width)^2^ × length × 0.5. The DeadEnd™ Colorimetric TUNEL System (Promega) was used to assay apoptosis. For human NK cell detection, immunohistochemistry was performed using a mouse monoclonal antibody against CD56 (Dako). Numbers of human NK cells or TUNEL-positive cells were counted in 5 randomly selected fields for each mouse.

### Statistical analysis

Data are presented as mean + standard deviation (SD) or standard error (SE). Statistical comparisons of groups were performed using the Student’s t test. Values of *P <0.05* were considered to be statistically significant.

## Results

### Spontaneous lung metastasis of patient-derived GBM cells in orthotopic xenograft animal models using NSG mice

**Fig. 1:**
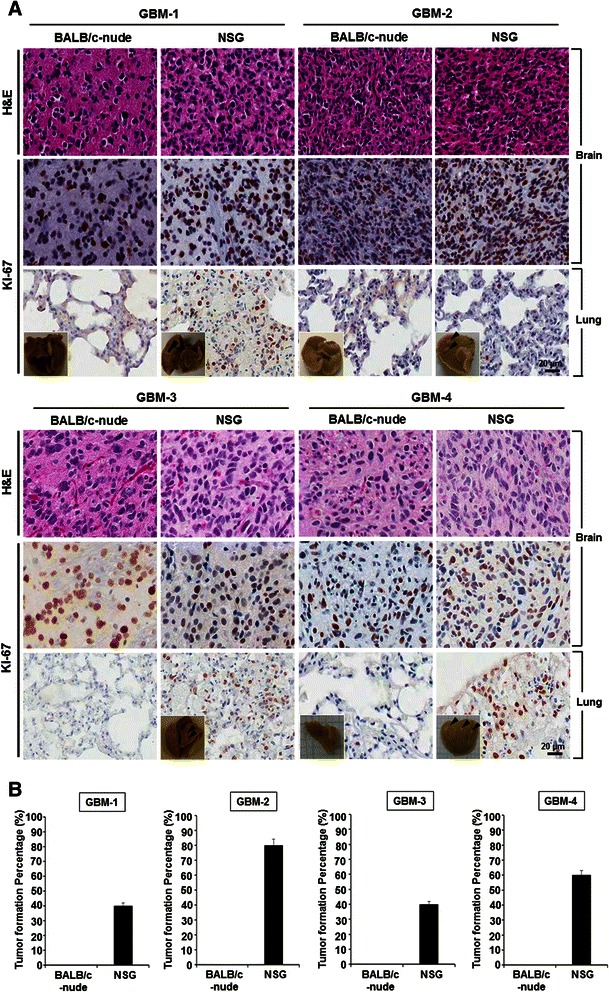
Brain and metastatic lung tumor formation in an orthotopic xenograft animal model using patient-derived GBM cells. **a** Pathologic validation of brain and metastatic lung tumors in various orthotopic xenograft animal models (*n* = 5 for each group). Tumor cells were transplanted into the mouse brain parenchyma. Immunohistochemistry against a cell proliferation marker (Ki-67) was performed. **b** Metastatic lung tumor formation rate was compared between BALB/c-nude and NSG recipient mouse strains

One of issues that could increase the translational value of the orthotopic xenograft model using patient-derived GBM cells, is an experimental protocol that would improve the in vivo tumor-take rate and shorten the latent period for the formation of detectable xenograft tumors. We hypothesized that recipient mouse strains with different levels of immune deficiency could be independent experimental variables that make a difference in the establishment of a GBM orthotopic xenograft model. Based on the hypothesis, we adopted two immune deficient mouse strains, BALB/c-nude and NSG mice, and then four types of patient-derived GBM cells were stereotactically injected into the mice’s brains (Table 1). Compared with the BALB/c-nude strain, NSG mice have a greater immune deficiency, including an impaired innate immune system [16]. In vivo tumorigenicity was defined as the formation of a tumor within the 6 months after tumor cell transplantation [13]. Overall in vivo tumor-take rates were not different between the BALB/c-nude and NSG groups (Table 1). However, median survivals of the NSG groups were significantly shorter than those of the BALB/c-nude groups (*P < 0.001* for GBM-1, GBM-2, and GBM-3, *P < 0.01* for GBM-4, Table 1), which indicates that the level of immune deficiency could have an effect on the orthotopic in vivo tumor formation of patient-derived GBM cells. Orthotopic xenograft tumor formation was confirmed by the pathology and immunohistochemistry against the proliferation marker, Ki-67 (Fig. 1a).

**Table 1:** In vivo tumor formation rate and median survival length of orthotopic GBM xenograft animal models

Patient.no.	Mouse	Mouse number (Tumor formation/Total)		Median survival day
		Brain	Lung	
GBM-1	BALB/c-nude	4/5 (80 %)	0/5 (0 %)	206
	NSG	4/5 (80 %)	2/5 (40 %)	176***
GBM-2	BALB/c-nude	4/5 (80 %)	0/5 (0 %)	130
	NSG	3/5 (60 %)	4/5 (80 %)	91***
GBM-3	BALB/c-nude	3/5 (60 %)	0/5 (0 %)	145
	NSG	3/5 (60 %)	2/5 (40 %)	121***
GBM-4	BALB/c-nude	4/5 (80 %)	0/5 (0 %)	140
	NSG	4/5 (80 %)	3/5 (60 %)	120**

**p < 0.01, ***p < 0.001

Unexpectedly, we detected abnormal respiratory movements in the NSG groups. Although extra-cranial metastasis of GBMs is extremely rare in GBM patients [7–9] the abnormal behavior observed suggested a spontaneous lung metastasis of orthotopically-implanted patient-derived GBM cells. When the lungs of both the BALB/c-nude and NSG groups were harvested, multiple visible lung nodules were observed only in the NSG groups (Table 1, arrowheads in Fig. 1a). The oncologic origin of the nodules was confirmed by the pathology and immunohistochemistry against Ki-67 (Fig. 1a). The lung nodules in the NSG mice were consistently detected in all four kinds of patient-derived GBM cells (Fig. 1b). Compared with the BALB/c-nude mouse, the NSG mouse strain has severe impairments, not only in adaptive T- and B-cell activities but also in innate immunity, involving macrophages and natural killer (NK) cells. Since metastasizing GBM cells meet circulating immune cells in the blood stream, we hypothesized that presence of NK cells in the circulating system might cause those sharp differences in the extra-cranial metastatic potential of implanted patient-derived GBM cells.

### Spontaneous extra-cranial metastasis of GBM cells provoked by NK cell-inactivation in BALB/c-nude mice

**Fig. 2:**
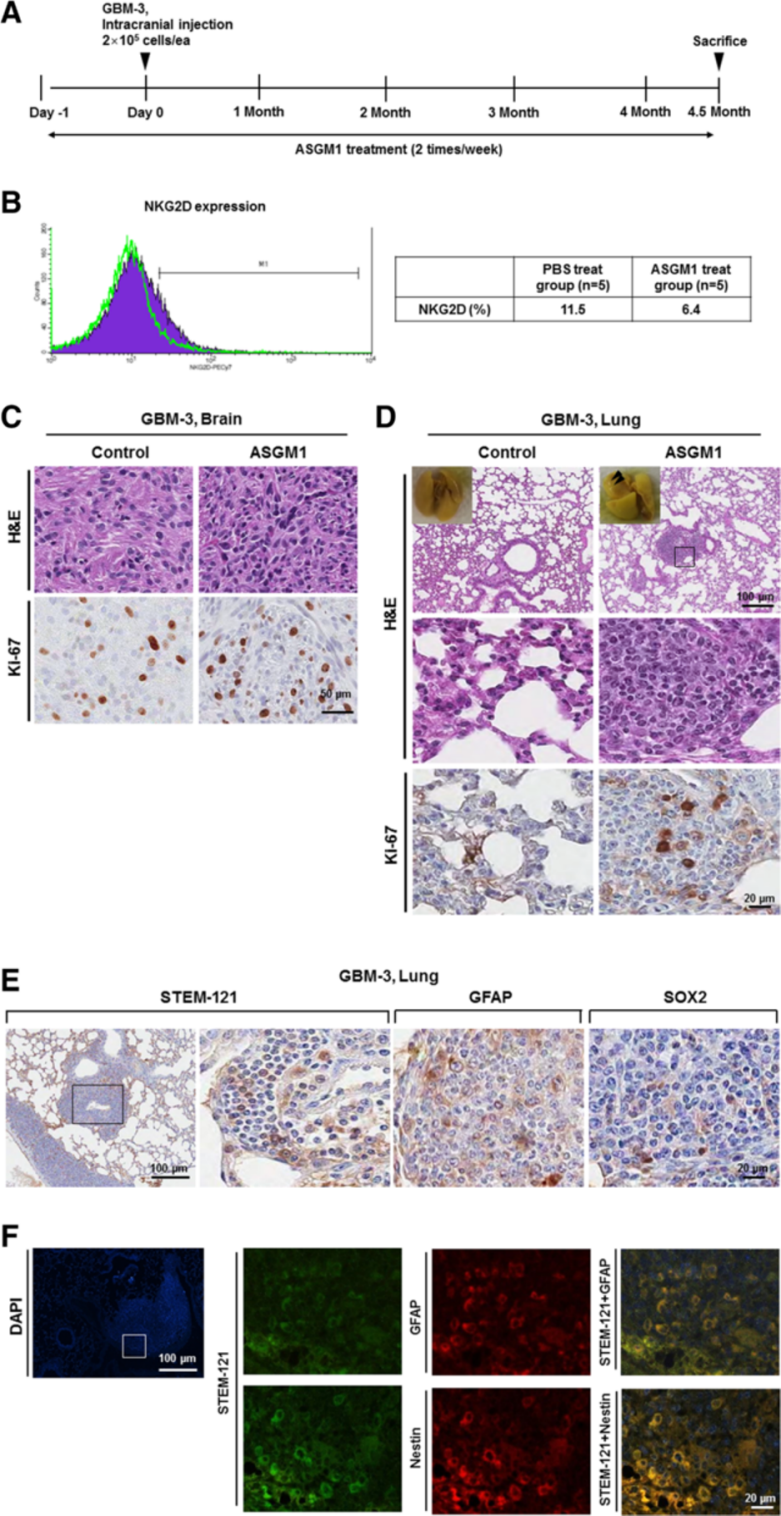
NK cell inactivation provokes spontaneous lung metastasis of GBM cells in the brains of BALB/c-nude mice. **a** Experimental schedule illustrated. **b** Murine splenic NK cells were analyzed by flow cytometry. NKG2D-posive cell ratio was analyzed and compared. **c**, **d** Pathologic validation of brain (**c**) and metastatic lung tumors (**d**) in various orthotopic xenograft animal models. Immunohistochemistry against a cell proliferation marker (Ki-67) was performed. **e** Immunohistochemistry against human specific cytoplasmic antigen (STEM-121) and GBM specific markers (GFAP, Nestin and SOX2) in metastatic lung tumors. **f** Human-specific cytoplasmic antigen (STEM-121) was co-localized with GBM specific markers (GFAP or Nestin)

To evaluate our hypothesis, we inactivated NK cells in BALB/c-nude mice using an activated NK cell-inhibition ASGM1 antibody [19–21] and then orthotopically transplanted patient-derived GBM cells (2 × 10^5^/ea, GBM-3) (Fig. 2a). ASGM1 treatment (2 times/week) was maintained for 4.5 months, the median survival time of BALB/c-nude orthotopic xenograft animal model using GBM-3 cells (Table 1). ASGM1-treated mice showed significantly fewer NKG2D-positive NK cells in the spleen than PBS-treated control mice, indicating the successful inactivation of systemic NK cells (Fig. 2b). When the control and NK cell-inactivated mice were sacrificed simultaneously (Fig. 2a), orthotopic brain tumor take-rates were not different (Fig. 2c, *n* = 5 for each group). In contrast, lung nodules were observed only in the ASGM1-treated mice (Fig. 2d, 60 % of the treated mice). The oncologic origin of the nodules was confirmed by the pathology and immunohistochemistry against Ki-67 (Fig. 2d). We further performed immunohistochemistry against human specific cytoplasmic antigen (STEM-121) and GBM specific markers (GFAP, Nestin, and SOX2) to confirm that the lung nodules were composed with human GBM cells. Cells in the lung nodules expressed all human-specific or GBM-specific antigens (Fig. 2e), and the antigens were co-localized in same cells (Fig. 2f). These results indicate that NK cells are responsible for the inhibition of extra-cranial metastasis of patient-derived GBM cells in BALB/c-nude mice. Although it is not included in this study, it need to be elucidated further whether NSG mice with GBM cells in their brains have fewer lung metastases when they are supplemented with NK cells.

### NK cell distribution in orthotopic GBM xenografts and MHC class I molecule expression of patient-derived GBM cells

**Fig. 3:**
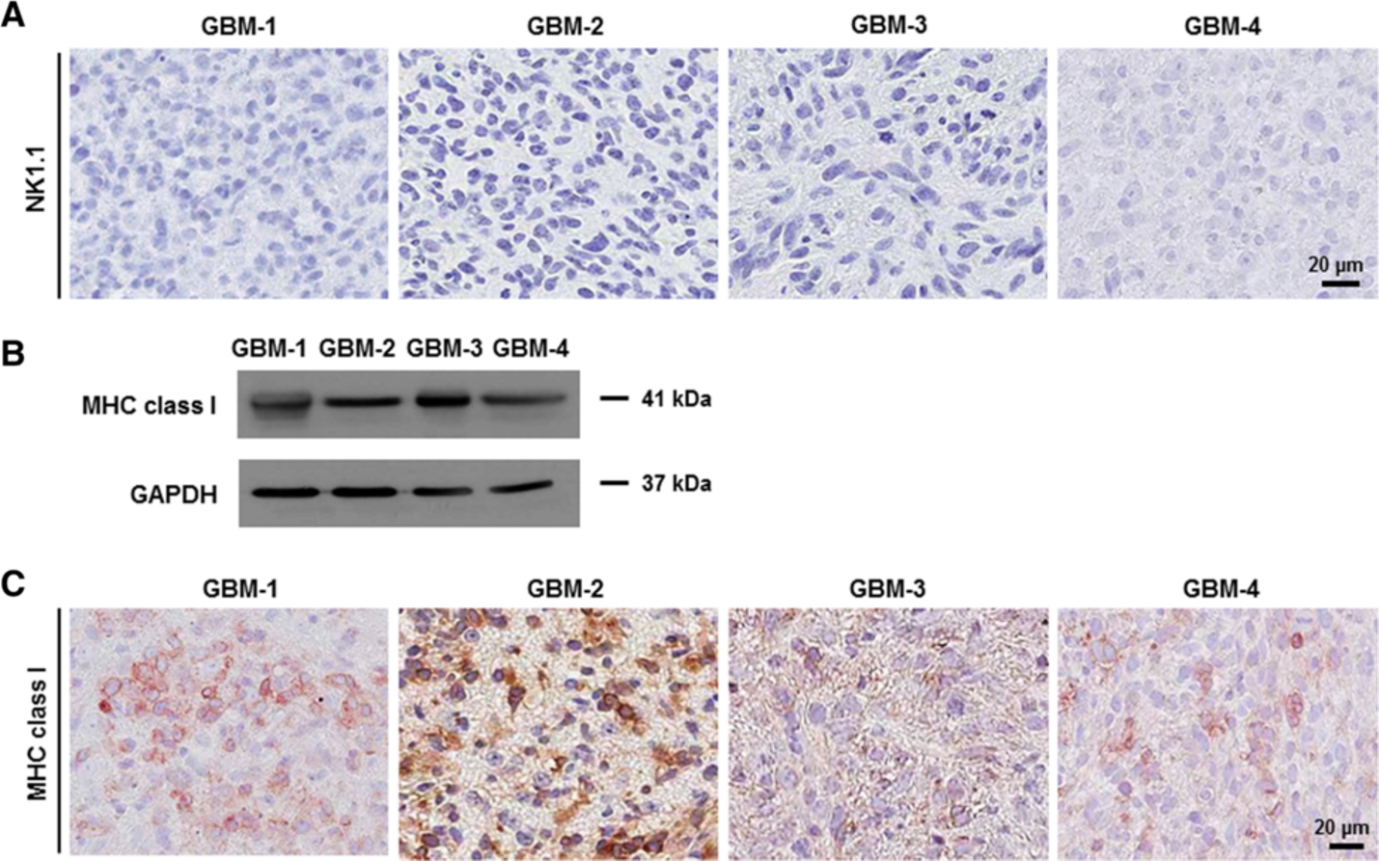
Expression of NK1.1 and MHC class I molecules in orthotopic GBM xenograft tumors. **a** NK cells were immunostained with a specific antibody against NK1.1 in the orthotopic GBM xenograft tumors. **b**, **c** MHC class I molecule expression of patient-derived GBM cells were analyzed by western blotting (**b**) and immunohistochemistry in the orthotopic GBM xenograft tumors (**c**). GAPDH = loading control

NK cells affected the extra-cranial metastasis of patient-derived GBM cells in BALB/c-nude mice but not in orthotopic brain tumor formation. NK cells have been suggested to be involved in xenograft rejections although their molecular mechanism has yet to be elucidated [14, 22]. Since the brain is an immunologically privileged organ [23], the absence of NK cells in the brain could result in the extra-cranial metastasis-specific effects. When we performed immunohistochemistry against NK1.1, a NK cell marker, in the orthotopic xenograft tumors, NK1.1-positive cells were hardly observed in the BALB/c-nude mice (Fig. 3a). We also evaluated MHC class I molecule expression of patient-derived GBM cells in the orthotopic xenograft animal model, since MHC class I molecules give inhibitory signals to NK cells [1, 24, 25]. Although patient-derived GBM cells expressed MHC class I molecules, the expression was very heterogeneous; as some cells did not show immunoreactivity to the antibody against MHC class I molecules (Fig. 3b, c).

The absence of NK cells in brain tumor and heterogeneous MHC class I molecule expression of GBM cells suggest followings. First, engraftment of patient-derived GBM cells could be more successful in the brain than other organs that have reactive NK cells. Second, circulating NK cells could target metastasizing GBM cells in extra-cranial sites. Previously, invasive GBM cells were observed to lose expression of MHC class I molecules [26], which would increase the possibility that NK cells might recognize invasive and/or migrating GBM cells.

### Applicability of NK cell therapy to GBM

The absence of NK cells in brain tumors and the anti-cancer effects of NK cells inversely indicate that supplementation of reactive NK cells to GBMs could have therapeutic effects. To evaluate the possibility, we transferred NK cells to an orthotopic xenograft GBM animal model and its examined therapeutic effects. In the clinic, mouse NK cells could not be hired to treat human GBMs. To address this issue, human NK cells were utilized for the experiments.

### Large-scale expansion of human NK cells

**Fig. 4:**
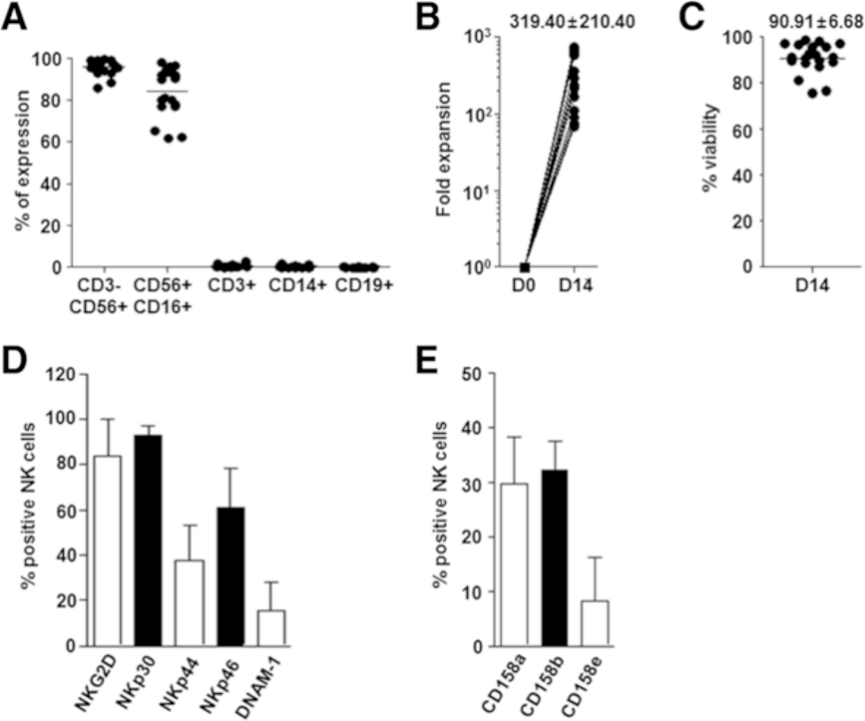
Large-scale in vitro expansion human NK cells and their phenotypic characteristics. **a** The expanded human NK cells were analyzed for various immune cell markers (*n* = 22). **b** The fold expansion of NK cells was determined by comparing the numbers of NK cells in the culture before (D0) and after (D14) the expansion (*n* = 20). **c** The viability of expanded NK cells was measured through propidium iodide staining (*n* = 20). Surface expression of activating (**d**) and inhibitory (**e**) receptors on expanded NK cells was analyzed by flow cytometry (*n* = 13). Data = mean + SD for (**d**) and (**e**)

Human peripheral blood mononuclear cells (PBMCs) were isolated from healthy volunteers, and then CD3 positive T cells were depleted by MACS using an anti-CD3 antibody. The resulting T cell-depleted PBMCs were co-cultured with irradiated autologous PBMCs in the presence of IL-2 and OKT-3 for 14 days. The expanded cells were composed of highly enriched CD3^-^CD56^+^ (95.74 ± 3.48 %) or CD56^+^CD16^+^ (84.05 ± 11.61 %) NK cells with minimal contamination by CD3^+^ T cells (0.59 ± 0.72 %), CD14^+^ monocytes (0.36 ± 0.51 %), and CD19^+^ B cells (0.12 ± 0.13 %) (Fig. 4a). During the culture, NK cells substantially expanded 319.40 ± 210.4-fold (Fig. 4b) with had a viability of 90.91 ± 6.68 % (Fig. 4c). Expanded NK cells showed high-level expression of activating receptors such as NKG2D, NKp30, NKp44, NKp46, and DNAM-1, suggesting their potent effector functions (Fig. 4d). Furthermore, expression of inhibitory KIR receptors (CD158a, CD158b, and CD158e) indicated that the expanded NK cells were sufficiently matured (Fig. 4e).

### In vitro GBM cell lysis effects of expanded NK cells

**Fig. 5:**
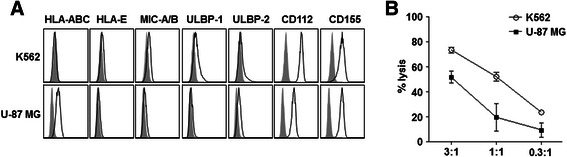
GBM cell lysis effects of expanded human NK cells. **a** Surface expression of HLA-ABC, HLA-E, NKG2D ligands (MIC-A/B, ULBP-1, and ULBP-2), and DNAM-1 ligands (CD112, CD155) on tumor cells was analyzed by flow cytometry. **b** In vitro cytotoxicity of expanded NK cells against K562 and U-87 MG cells was determined by ^51^Cr-release assay. Data = mean ± SD

Cytotoxic activity of expanded NK cells against human GBM U-87 MG and human immortalized myelogenous leukemia K562 (positive control) cells was evaluated in vitro. Compared with K562 cells, U-87 MG cells expressed MHC class I (HLA-ABC) molecules highly (Fig. 5a). However, U-87 MG cells also have high levels of NK cell-activating ligands such as CD112 and CD155 (DNAM-1 ligands) (Fig. 5a). When U-87 MG or K562 cells were co-cultured with expanded human NK cells (NK cell:tumor cells = 3:1, 1:1, or 0.3:1), NK cells lysed cancer cells in a dose-dependent manner (Fig. 5b). Since U-87 MG GBM cells express NK cell-inhibiting MHC class I molecules and have less NK cell-activating ligands (Fig. 5a), the cytotoxicity of NK cells was more prominent in K562 cells (Fig. 5b). These results suggest that GBM cells have a susceptibility to NK cells and that the balance between the inhibitory and activating signals for NK cell could be an important determinant of NK cell effects on target cells.

### In vivo therapeutic effects of NK cells against orthotopic GBM xenograft tumors

**Fig. 6:**
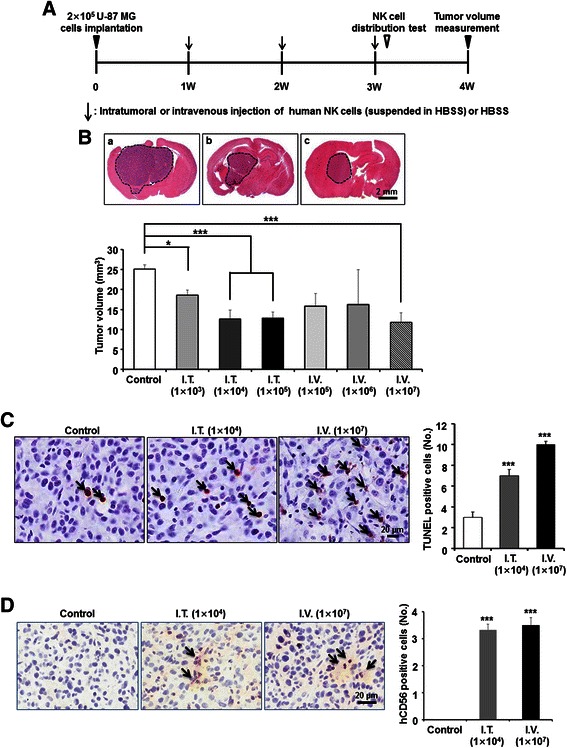
In vivo therapeutic effects of human NK cells against orthotopic GBM xenograft tumors. **a** Experimental schedule illustrated. **b** Tumor volumes were determined and compared one week after the last NK cell injection (*n* = 7 for each group). I.T. = intratumoral transplantation, I.V. = intravenous injection. Data = mean + SE. *p < 0.05, ***p < 0.001, vs. control. **c** Apoptotic tumor cells in the xenograft tumors were analyzed by the TUNEL assay. TUNEL-positive cells were calculated and compared with the control group. Arrow = TUNEL positive cell, Data = mean + SE. ***p < 0.001, vs. control. **d** Human NK cells were traced by immunohistochemistry using a CD56-specific antibody (*n* = 3 for each group). Number of NK cells were calculated and compared with the control group. Arrow = CD56 positive cell, Data = mean + SE. ***p < 0.001, vs. control

Next, the in vivo therapeutic efficacy of NK cells against GBM was examined. NK cells were injected intratumorally (1 × 10^3^, 1 × 10^4^, or 1 × 10^5^ in 5 μl HBSS) or intravenously (1 × 10^5^, 1 × 10^6^, or 1 × 10^7^ in 100 μl HBSS) into BALB/c-nude mice bearing U-87 MG tumors in their brains once a week for three weeks (control = intravenous HBSS only, each group *n* = 7; Fig. 6a). When tumor volumes were measured one week after the last NK cell injection, the intratumoral NK cell injection groups showed significantly smaller tumors in a dose-dependent manner. Compared with the control group, the 1 × 10^3^, 1 × 10^4^, and 1x10^5^ intratumoral injection groups showed 36, 57, and 56 % tumor volume reduction, respectively (Fig. 6b). When NK cells were injected intravenously, the 1 × 10^7^ intravenous injection group showed a 60 % reduction in tumor volume (*P < 0.001* vs. control), while the other intravenous injection groups had no statistically significant treatment effects (Fig. 6b). Significant increase in the numbers of TUNEL-positive apoptotic cells (Fig. 6c) were confirmed by immunohistochemistry in the 1 × 10^4^ intratumoral and 1 × 10^7^ intravenous injection groups. These results suggested that in vivo treatment of supplementation of NK cells has positive effects against orthotopic GBM xenograft tumors. Although fewer number (1 × 10^4^) of NK cells were intratumorally injected compared with the intravenous transplantation group (1 × 10^7^), similar numbers of NK cells were observed in the orthotopic GBM xenograft tumors (Fig. 6d) 24 h after the transplantation of NK cells (Fig. 6a). Since the two groups had similar treatment results (Fig. 6b), these results indicated that direct cell-to-cell interaction induced by infiltration of NK cells into the tumors in the brain parenchyma is required for the cytotoxic effects of NK cells.

### Implications of treatment schedule in the NK cell therapy against GBM

**Fig. 7:**
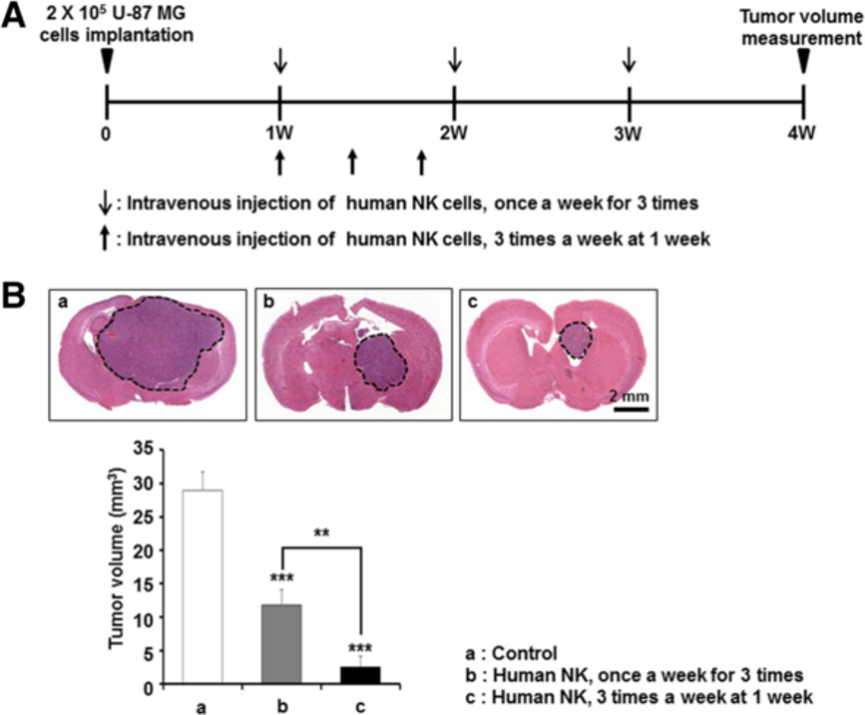
Effects of treatment schedule on the treatment effects of intravenous supplementation of Human NK cells. **a** Experimental schedule illustrated. **b** Tumor volumes were determined and compared four weeks after the tumor cell transplantation (*n* = 7 for each group). Data = mean + SE. ***p < 0.001, vs. control, **p < 0.01, vs human NK cell treated once a week for three weeks

Although the intratumoral injection of NK cells needed fewer cell numbers for in vivo treatment results, intravenous injection is more clinically applicable. To increase the NK cell-cancer cell interaction of intravenous treatment, we modified the treatment schedule from 1 × 10^7^ NK cells once a week for 3 weeks to 1 × 10^7^ NK cells three times a week for a week (Fig. 7a). The concentrated treatment would increase NK cell/tumor cell ratio in the brain tumor. Compared with once a week schedule (60 % tumor volume reduction compared with the HBSS intravenous injected control group), the three times a week treated group showed better treatment results (86 % tumor volume reduction) (Fig. 7b, *n* = 7 for each group). Therefore, optimized treatment schedules would potentiate the therapeutic effect of intravenous NK cell supplementation treatment against GBM.

## Discussion

In this study, we observed the spontaneous systemic metastasis of patient-derived GBM cells that were inoculated into the brains of NSG mice. Since the systemic metastasis was not induced in the BALB/c-nude mice, we hypothesized that the difference originates from the innate immune system such as NK cells, macrophages, and the complement system [14, 16, 22]. When we administrated murine NK cell inhibition antibody to BALB/c-nude orthotopic GBM animal models, extra-cranial metastases were induced. These results suggest that NK cells play an important inhibitory role in the systemic metastasis of GBMs and that GBM cells are sensitive to the cytotoxic activities of NK cells.

Traditionally, the brain has been regarded as an immunologically privileged site [2, 4, 5, 27, 28]. In orthotopic GBM xenograft tumors, NK cells were not observed by immunohistochemistry in this study. NK cells identify and eliminate cells lacking MHC class I molecules, which are dependent on the activation status of NK cell-activating and inhibitory receptors [29–33]. We observed that the expression of MHC class I molecules in patient-derived GBM cells is heterogeneous. However, ly49 receptors of mouse NK cells could not recognize human MHC class I molecules [34–37]. Therefore, NK cells of BALB/c-nude mice would target human patient-derived GBM cells, while human patient-derived GBM cells in the brain parenchyma could not have direct contacts with NK cells.

In vitro expanded human NK cells showed cytotoxic effects against human U-87 MG GBM cells in vitro and in vivo, although U-87 MG cells expressed NK cell-inhibiting MHC class I molecules (HLA-ABC). These unexpected results might originate from the expression of NK cell-activating ligands of U-87 MG cells such as ULBP-2, CD112, and CD155. Cytotoxic effects of NK cells are determined by the balance between NK cell-activating and inhibiting signals; when the activating signals are dominant, NK cells have cytotoxic effects [30, 33, 38]. Since human leukemia K562 cells do not express MHC class I molecules, NK cells showed superior killing effects to K562 cells compared with U-87 MG cells in this study.

Moreover, distribution of NK cells in vivo could be an important determinant of their therapeutic effects. In this study, we observed that the treatment efficacy of intratumoral injection of NK cells is better than intravenous injection; 1 × 10^4^ NK cells transplanted intratumorally showed similar therapeutic effects to 1 × 10^7^ intravenously transplanted NK cells. When we compared NK cell distribution in the orthotopic GBM xenograft tumors, similar densities of NK cells were observed in the 1 × 10^4^ intratumor and 1 × 10^7^ intravenous groups. Ratios of TUNEL-positive apoptotic cells to CD56 positive NK cells in the tumor (2 ~ 3:1) was also similar in those groups. Since NK cells do not distribute in the brain, techniques that increase the NK cell number in the GBM would increase the therapeutic effects of NK cells against the intracranial tumor. Accordingly, when NK cells were intravenously transplanted more intensively to increase the NK cell/tumor cell ratio, the treatment effects of NK cells were significantly potentiated in this study.

Immunotherapies such as dendritic cells, lymphocytes, engineered T-cells, and cancer vaccines, have shown treatment effects on patients with various metastatic solid tumors [39–42]. Cellular immunotherapeutic strategies directly target metastatic cancer cells combined with cytotoxic agents or ionizing radiation [43–46]. Moreover, tumor initiating/cancer stem cells with antigenic heterogeneity were suggested to be recognized by immune cells [47, 48]. Given that extremely rare extracranial metastasis of GBM [49] and sparse distribution of NK cells in the brain, inhibitory effects of NK cells on systemic metastasis of GBM cells in vivo in this study might be mediated by direct interaction between NK cells and GBM cells in the extracranial sites where NK cells monitor abnormal cells.

Recent successes of immunotherapy for solid tumors have generated a resurgence of interest in immunological therapeutic approaches to GBMs [23, 27, 38, 50–54]. Many immune cell-based therapies have been tried for the GBM, including dendritic cells, lymphokine-activated killer cells, NK cells, and cytotoxic T lymphocytes [32, 55]. Among them, NK cells have the strongest cytotoxic activities against malignant tumor cells and play important roles in the initiation of subsequent adaptive immunity [35, 36, 56]. In this study, we generated sufficient human NK cells in vitro and confirmed their direct-tumor killing effects against GBM cells both in vitro and in vivo. Moreover, other immune cells, such as mouse macrophages, could be recruited to the GBM by the NK cell therapy, which would provoke further immunological reactions to GBM cells.

## Conclusions

In summary, our results suggest that NK cells play an important inhibitory role in the extra-cranial metastasis of the GBM and that GBM cells are susceptible to NK cells. Therefore, supplementation of NK cells is a promising immunotherapeutic strategy against GBM, which could be potentiated by techniques that augment direct cell-to-cell contact between GBM and NK cells.

## Additional file

Additional file 1: **The ARRIVE Guidelines Checklist Animal Research: Reporting In Vivo Experiments.** (PDF 813 kb)
